# Illinois State Medical Society

**Published:** 1888-06

**Authors:** 


### ILLINOIS STATE MEDICAL SOCIETY.

Thirty-eighth Annual Meeting, held at Rock Island, Illinois, May 15, 16, and 17.

The Society was called to order at 10 o'clock by the President, Dr. W. O. Ensign, of Rutland.

ADDRESS OF WELCOME.

Dr. C. Truesdale, of Rock Island, chairman of the committee of arrangements, delivered an address of welcome on behalf of the local profession.

Professor C. W. Earle, of Chicago, responded in behalf of the visiting members of the Society.

The Secretary read the resignation of the Treasurer, Professor Walter Hay of Chicago.

On motion, the thanks of the Society were tendered to Professor Hay for his very efficient services as Treasurer.

The President, Dr. Ensign, in his address, paid a tribute to those who had preceded him in office and alluded to the large number of deaths that had occurred during the year among members.

He then dealt with the question of reorganization, the plans and modes of holding the annual meetings, etc. He thought the meeting of the State Society, occurring so soon after that of the American Medical Association, detracted largely from its interest and attendance. The address referred to the relation of the State Board of Health to the study and practice of medicine in the State, favoring the limitation of the issue of certificates to practice to those only who could pass a successful examination before a State board of examiners. More thoroughness in all the preliminary branches of education was urged.

COMMITTEE ON PRACTICE OF MEDICINE.

The chairman of the committee on practice of medicine, Professor Frank Billings, of Chicago, said: *Bergeon's treatment of phthisis pulmonalis*, by rectal injections of sulphuretted gas in carbonic acid, received extended notice and discussion at the last meeting of the Society. The expectations regarding the remedy have not been realized. The treatment has been thoroughly tested in the United States, and the almost universal opinion seems to be that it exerts no curative influence on the tubercular process, but does have a palliative effect; diminishing the sputa; relieving the cough, nightsweats, and thoracic pain; improving the appetite, and consequently the strength of the patient. These favorable conditions are only temporary, however, and a time comes when the injections cease to have a good effect.

*The aniline treatment of phthisis*, recommended by Dr. Kremianski, consists of the internal use of grammes .5 to 1 daily of antifebrin, which is decomposed in the body into aniline, and also of inhalations of oil of aniline in one of the aromatic oils. Dr. Bertalero reports successful results in eight cases. Professor Billings had used the treatment in two cases with absolutely no benefit.

Concerning *treatment of diphtheria by the use of oil of turpentine*, he said a new impetus had recently been given to it. Dr. C. Roesse, of Hamburg, had reported its use in 60 patients, from 1 to 30 years of age. Only five died. Of these, two were one year old, were moribund when first seen, and died within a few hours. Two had the disease in a virulent form and lived only thirty six hours, and the fifth was a poorly nourished boy, 15 years old, who died in five days. The oil of turpentine was given in teaspoonful doses three times a day. As a corrective Dr. Roesse used spiritus ætherus, minims xvi, to each teaspoonful of turpentine oil. He also gave a tablespoonful of a two per cent. solution of sodium salicylate every two hours, had the patient use a gargle of a one per cent. solution of potassium chloras, and applied ice-bags. He noted the following results: (1) The fever and frequency of the pulse diminished rapidly; (2) the subjective difficulties, such as headache and difficulty of swallowing, were soon relieved; (3) the duration of the disease was greatly shortened; (4) the local symptoms improved, in most cases as soon as the first doses had taken effect. The turpentine was used cautiously in anæmic and weak patients, and cases of cardiac troubles. In strong patients the heart's action was carefully noted. The patients were given strong bouillon or beef tea, port wine, and milk. Ice-water was given for thirst. The turpentine was withheld as soon as the patient was free from fever and the local symptoms had improved. No signs of turpentine poisoning were observed.

Professor Billings stated that he has had gratifying success with a *solution of corrosive sublimate*, one part to 1500 of water, on the local symptoms, as a gargle, or with a syringe with a spray nozzle, as was first suggested to him by professor S. Johnson, of Chicago. The spray may be injected with sufficient force against the pharyngeal walls and tonsils to dislodge the membrane and yet not wound or irritate the abraded surfaces.

Of *saccharine in diabetes mellitus*, he said: This valuable addition to our materia medica renders the treatment of diabetes much easier. He had used it in three cases of simple glucosuria and in one case of diabetes mellitus. It fills the place of sugar as a sweetening agent, and also acts well as an antiseptic in preventing or relieving gaseous distension of the stomach and bowels, which are often present in diabetes mellitus.

ANTIPYRIN.

Every day adds a new and valuable use of this remedy. Professor See first used it hypodermically in watery solutions in all forms of neuralgia, rheumatism, and other painful affections. He advises friction over the injected area to relieve the disagreeable tension.

Dr. R. Hirish reports the use of the hypodermic injection of a 50 per centum solution in twelve cases of rheumatism with marked success, relieving the pain and shortening the course of the disease.

ANTIFEBRIN.

Dr. Dumieville has used antifebrin as an anodyne in all neuralgias in over eighty cases with universally good effect. Fifteen grains in twenty-four hours was the average dose. The effect is felt in from fifteen minutes to two hours. Several reports have been made of cases of dangerous collapse, with or without cyanosis, from the use of the drug. One death is reported of a child from the use of antifebrin. The father of the child doubled the dose ordered by by the physician, and death occurred from heart failure in twelve hours.

SULPHATE OF SPARTEIN.

This remedy has received more extended use during the last year, and many favorable notices of it have been written. Dr. J. Prior reports that it is indicated in all diseases of the heart where digitalis is contraindicated, where a quick effect is desired, and as an adjuvant of digitalis. He gives the remedy in doses of .01 to .02 grammes two to three times daily, and says the usually advised dose is too small.

DISCUSSION.

Dr. C. TRUESDALE, of Rock Island, said that his experience and observations in the treatment of diphtheria were that the main supports depended upon the character of the case he had to treat.

He had used turpentine, bichloride of mercury, etc. He believed that the best remedy was the old-fashioned treatment of chlorinated tincture of iron in large doses, and it was remarkable what large doses young children would tolerate of that drug.

Professor C. W. EARLE, of Chicago, said that it had always appeared to him, particularly in children, that simply catching cold, or exposure to it, could not account for a very large number of cases of pneumonia reported. He believed that there must be something beyond that--something in the earth or atmosphere to produce pneumonia in a large number of cases, although he had never conducted any scientific experiments to base such conclusions upon; but it was on this account that he always administered quinine in the treatment. He now regarded pneumonia as a very safe disease in a child, and much preferred a case of it to one of bronchitis involving the small tubes.

With regard to diphtheria, he uses the tincture of iron and chlorate of potash once in every four hours, but where it is desirable to make a strong impression upon the local disease he has given that mixture every two hours, and between it minute doses of hydrargyrum bichloride.

Dr. J. W. COWDEN, of Rock Island, said that Dr. Jacobi recommends tincture of iron and chloride of potassium. He, himself, gave it every fifteen minutes in teaspoonful doses to an adult, and to a child two years of age ten drops of tincture of iron. It would allay the pharyngeal inflammation, and takes the place of a gargle.

Another thing was to support children by giving them milk in abundance, and alcohol when it is well borne. He had a little child last winter that took a teaspoonful of whisky every half-hour for forty-eight hours (warm), and would take nothing else in it.

Professor HENRY J. REYNOLDS, of Chicago, asked for some further light in regard to the physiological effects of antipyrin. He said it was an extremely powerful drug and as useful as it is powerful, but thought it capable of doing a great deal of harm, in that in some cases it produced disorder of the kidneys, suppression of the urine, uræmic poisoning, etc.

Dr. T. M. McILVAINE, of Peoria, had used turpentine in the treatment of diphtheria in much larger doses than those mentioned by any of the previous speakers and had had no toxic effects from the drug.

He had great confidence in bichloride of mercury, used both internally and as a spray. He gives it in combination with lactic acid as a spray.

The conditions under which antipyrin is administered should be more carefully studied. On some patients it acted admirably under certain conditions, and again in the same patients the same dose had produced very unpleasant symptoms. He had in mind the case of a lady who had taken it several times with almost immediate relief, in seven and a half grain doses, repeated in one hour if the first dose was not sufficient. But on another occasion, when the drug was made use of, it brought on unpleasant symptoms, such as cyanosis, dizziness, trembling, purple lips, and purple finger nails.

Dr. J. S. MILLER, of Peoria, remarked that in the treatment of diphtheria it seemed to him that, as Dr. Truesdale had said, we should adapt our remedies to meet the indications and emergencies of our cases.

He had used antipyrin in one case of typhoid fever, and it produced almost fatal collapse. He had recently treated a case of malarial neuralgia with most excellent effect with antipyrin; a few doses controlled it immediately.

Dr. J. W. MORGAN, of Port Byron, had used the biniodide of mercury in the treatment of diphtheria with gratifying results. It was far better than corrosive sublimate from the fact that it was the most diffusable mercurial preparation we have.

Professor BILLINGS, in closing the discussion, said the paper was not intended to embrace the treatment of diphtheria, phthisis pulmonalis, or anything else, but was simply a résumé of the progress made in medicine during the year.

### COMMITTEE ON GYNÆCOLOGY.

Professor A. Reeves Jackson, of Chicago, chairman of this committee, read a paper entitled "A Plea for Conservatism in Gynæcology," which appears in another part of this journal.

Dr. T. M. Cullimore, of Jacksonville, read a paper on the "Menopause and the Pathological Conditions Incident to it."

### DISCUSSION.

Dr. O. B. WILL, of Peoria, in opening the discussion, said that so far as he was personally concerned he could indorse every sentiment expressed in Professor Jackson's able paper. He could not agree with Dr. Cullimore, however, that the menopause, or change of life, was pathological. It is a physiological change, and as such, under circumstances of good general health, will terminate favorably in each instance.

Professor C.W. Earle, of Chicago, said, in commendation of Professor Jackson's paper, that it was timely and exceedingly appropriate, and he thought it absolutely necessary that some one should read just exactly such a paper as Professor Jackson's with the hope of eradicating or removing the craze that had seized a large number of physicians for operating.

Dr. C. Truesdale, of Rock Island, remarked that he may have misunderstood Dr. Jackson in the beginning of his paper as to whether he commended the work of Lawson Tait and Sir Spencer Wells. If he understood the matter aright, a laparotomy that had for its object the removal of the uterine appendages was an operation that was being greatly abused, and that if there was any man responsible for it, it was Lawson Tait, for he had operated in this way oftener than any other man in the civilized world. He would admit that Tait had been sucessful in his operations, if the reports of percentages of death could be relied upon.

Professor Jackson, in closing the discussion, said that his allusion to the work of Sir Spencer Wells and Mr. Tait was only intended to show that, whether they were always right or wrong, there is a foundation for the great amount of work they had done and are doing, as he considered them honest, and highly commended them in such a line of work; but he had reference more particularly to the fact that, perhaps, hundreds of lives had been sacrificed through the indiscriminate use of the operation by inexperienced, ambitious imitators, whose desire was to open the abdomen and cut something out.

### Second Day—Morning Session.

### Antiseptic Obstetrics.

The chairman of this committee, Professor C. W. Earle, called attention to the following questions:

*First.* Does the high rate of mortality still remain in the private practice of obstetrics?

*Second.* Can the extreme low rate of mortality attained in hospital obstetric practice be obtained in private practice?

Not many years since Mr. Lawson Tait paid a visit to Professor Tarnier at La Maternite. The Professor called the attention of Mr. Tait to a linear chart on the wall of his room, showing the total death-rate of women confined in that hospital from 1792 to 1886. This record is divided into three periods: (1) That of inaction, in which the mortality was from 9.3 to 20 per cent.; (2) the battle of hygiene against infection and contagion, with a mortality of 2.3; (3) the victory of antiseptics, with a mortality of less than 1 per cent., and in the Tarnier pavillion, since June, 1880, with 785 deliveries, not a death has taken place.

About the year 1847 Semmelweiss wrote: "Puerperal fever has existed for two hundred years; it is time that it should disappear."

Concerning puerperal fever in ancient times we know but little. Litzman believes it should be classed among the modern diseases. But in 1864 it raged in the Hotel Dieu, in the Dublin Maternity in 1672, and again in 1774 in Paris and Dublin, and up to the end of that century in Vienna, Berlin, Giessen, Copenhagen, and St. Petersburg. In the last named place, in 1825, one in eleven died; in Lombardy, between 1786 and 1887, not a patient survived.

About 1880, Pasteur believed he saw the microbe of puerperal fever. Condensed from practical use, it has been demonstrated that (1) the air and water of the earth are crowded with organized microscopic beings; (2) they live and multiply at the expense of organized matter; (3) their penetration into the tissues produces disease; (4) the skin, respiratory and digestive passages furnish the channels into the body; (5) a healthy tissue has never produced a microbe; (6) any abrasion of tissue against which these microbes come makes it possible for them to enter the system; (7) unless germs are brought from without there can be no infection; (8) many things in regard to their virulence, nature, contagion, age, and development, are yet under consideration. The application of these principles has given the victory to antiseptics in hospitals.

As soon as it is known that confinement of a patient is about to take place, Professor Earle is in the habit of requiring her to take a warm bath, the lower part of her person is to be washed with carbolized water. It is also requested that one or two carbolized vaginal injections shall be taken during the first stage of her labor.

The nurse should subscribe to certain rules of cleanliness. The lying-in chamber should be in a room where no infectious diseases have been treated, and all bed clothing should be prepared by boiling in a given per cent. of carbolized water. Do not select a matress which is filthy for fear that a better and cleaner one will be soiled by blood and other discharges during confinement. Do not provide pieces of old comforters, the sanitary condition of which is problematical. Do not borrow syringe or bed-pan, and assure yourself that those you have are boiled or washed in hot water and thoroughly carbolized.

### Committee on Obstetrics.

Dr. George Wheeler Jones, of Danville, the chairman, said:

During the past year very little, if anything, of special importance has occured in this branch of medicine which could be called strictly new.

#### CÆSAREAN SECTION.

The propriety of this operation in a large proportion of cases usually relegated to craniotomy has been presented with arguments of so forcible a character as to demand the attention of every accoucheur. The line of reasoning in favor of the operation of abdominal section is directed largely toward the rights of the child. With the operation, as improved and modified by Saenger, the reduction of mortality to both mother and child has been so great as to make the preference for the abdominal section not a matter of question, but greatly one of choice where there is reason to presume that the child is living, and the slight resulting injuries to the mother in case of her presumable recovery would suggest its extreme propriety, if not preference, where the fœtus is dead.

#### CONSERVATIVE OBSTETRICS.

The tendency of the day and hour is more and more toward conservative obstetrics—place the patient in the best possible condition and let her alone. Crèdé takes such extreme grounds, and goes so far in this matter, that he makes his diagnosis of position, condition, and advancement of case by external palpation and manipulation of the uterus through the walls of the belly, leaving the genitalia entirely alone. That ordinary accoucheurs will ever reach this degree of skill, or that it is best they should, is open to very serious doubt. Indeed the amount of injury possible to both mother and child by careless external manipulation is too great to justify the efforts, except at the hands of those exceptionally skillful.

An adherence to the general rules of which Crèdé is the exponent, along with carefully inducted asepsis, will reduce the proportion of cases of puerperal fever to so small a minimum as to make it relatively scarcely worth considering.

#### TREATMENT OF EXTRA-UTERINE PREGNANCY.

The treatment of extrauterine pregnancy has been placed upon a firm basis from which there will not soon be any departure.

Electricity has robbed it of its terrors and placed it within the cure of readily curable conditions, for which physicians could not be too thankful. Eclampsia has another antagonist in the form of veratrum viride, which is being boldly advanced as a specific by several reliable practitioners. It must be given fearlessly in full doses. Its action is that of a vaso-motor paralyxant. Antipyrin and acetanilid, especially the latter, are growing in favor as reliable remedies for controlling many of the abnormal nervous manifestations of pregnancy. They control reflex irritability in a remarkable manner, and in the cephalalgia of the first stage, where there is convulsive action, Dr. Jones had been delighted several times with the action of acetanilid. He gives this agent in many cases where he formerly gave an opiate, and liked its action much better.

SECOND DAY—Afternoon Session.

Professor F. S. Johnson, of Chicago, one of the special committee to report on the *Present Status of Bacteriology in its Relation to Disease* read a paper on "The Bacillus in Syphilis."

Few questions, he said, in medicine have received more attention than that of the nature of the poison of syphilis. Early in this century the most able investigators had given it careful study, but not until within the last few years has there appeared to be any real advance in the knowledge of the subject.

In 1884 and 1885 Lusgarten found a bacillus which he describes as follows: In size and shape it very closely resembles the *tubercle bacillus* of Koch, and *lepra bacillus* of Hansen. It is sometimes bent at an angle, or is spoon-curved. In color reaction, it is also similar to the tubercle bacillus. It does not occur, however, in the tissue spaces, but is found in cells; very few are found in the lesions. They are found in all the lesions of syphilis primary, secondary, and tertiary, and are found in the syphilitic secretions.

Matterstock found, also, that the conditions best suited to the development of the smegma bacillus were (1) a mixture of the secretion from sebaceous glands and macerated epithelium; (2) equable temperature of about the body warmth, and (3) an acid reaction of the cultured medium.

SUMMARY.

1. A bacillus recognizable by the form, size, and color. Reaction has been found by a number of observers in all syphilitic lesions, and in all stages of the disease, and has also been found in the blood of syphilitic patients. There is a widespread belief among investigators of this subject that this organism has an etiological relation to the disease.

2. This bacillus is not uniformly found in the secretions of ulcerating, syphilitic lesions.

3. A bacillus almost identical in form, size, and color reaction has been found in the secretions of the external genitalia of healthy persons.

4. The possible presence of this smegma bacillus in the secretion of doubtful sores renders microscopical examination of the secretions in these cases nearly valueless.

COMMITTEE ON DERMATOLOGY AND VENEREAL DISEASES.

Professor H. J. Reynolds, of Chicago, referred first to a case of Tylosis of the Hands.

In an article on the uses of concentrated lactic acid, by Dr. J. P. Knoche (Jour. Cut. & Ven. Diseases, April, 1887), he states that he found that by rubbing in the undiluted acid for a few minutes and gentle scraping that much of the thickening could be removed. He again applied it seven hours afterwards, and in twenty-four hours was able to remove the thickening completely, leaving the skin soft and smooth. He then treated the parts with oxide of zinc and tar ointment, but found in about a week the growth began to reappear. The acid was again applied and repeated, using the above ointment at intervals, and after a few months of this treatment the parts remained cured. When applied two or three times daily it is a most efficient treatment for warts. In freckles and chloasma he found the acid when diluted to one-third its strength and applied daily to be the the most efficient means employed for that purpose.

CHANCROID.

In treating this affection he had used almost nothing in the way of medicine but pure impalpable boracic acid, ordering the patient to wash the parts in soap and water, three or four times a day, before applying the powder. The ulcers treated in this way would heal remarkably well.

Dr. G. W. JONES, of Danville, in opening the discussion, said he emphatically endorsed Dr. Reynolds' treatment of chancroid. He had found nothing so useful as impalpable powder of boracic acid in the treatment of such affections, more particular in women where the vaginal canal could be packed with it.

Dr. J. H. MILLER, of Oconee, said that in addition to boracic acid in gonorrhœa he combined with it the sulpho-carbolate of zinc with good effect.

Dr. MILLER, of Peoria, remarked that he had used boracic acid almost exclusively during the past three or four years in treating chancroids. The ulcers or sores had healed most kindly, and no destructive action had taken place in the cases which formerly gave him so much difficulty and annoyance.

In addition to pain, the patient sometimes complains of a sense of fullness or swelling, of dryness, burning, tickling, or itching, or simply of discomfort in the parts. There is neither fever nor acceleration of the pulse, excepting as a result of anxiety or alarm. Usually there is neither cough nor expectoration, but in certain localities the irritation may excite cough. The tongue may be slightly coated; but usually the appetite is good, and indeed the general condition is that of perfect health.

COMMITTEE ON OPHTHALMOLOGY AND OTOLOGY.

Dr. S. S. Bishop, of Chicago, read a paper on "A New Otoscope (pneumatic) for the Diagnosis and Treatment of Middle ear Affections under Passive Motion," and exhibited the instrument which he had devised for the purpose named. It consists of a small milled cylinder with an ear funnel of the most serviceable pattern at one end, and an eye piece containing a lens, around which revolves an adjustable mirror, at the other end. In the side of the cylinder an aperture admits light to the reflecting surface beneath. At the funnel end of the instrument is a pneumatic chamber provided with a rubber bulb with a flexible tube ending in a diminutive syringe, or a lip piece, as one may prefer.

He said the value of passive motion in the treatment of stiff joints and atrophied tissues is well recognized in general surgery. The application of the same principle to the same conditions in aural surgery is attended with equally beneficial results.

THIRD DAY—May 17.

Professor D. W. Graham, of Chicago, read a paper on "Some Points in Hernia Clinically Considered," in which he advocated the following points:

1. An umbilical or femoral hernia, with pronounced symptoms of strangulation, demands herniotomy without previous resort to taxis.

2. Irreducible omental hernia, with or without symptoms of strangulation, demands operation for the patient's safety.

The following papers were also read:

Professor E. Fletcher Ingals, of Chicago, read a paper on "Chronic Rheumatic Laryngitis, or Chronic Rheumatic Sore Throat," a painful affection of the throat, chronic in character, but varying much in severity from time to time, and attended by only slight physical changes in the parts involved. Usually some part of the larynx is involved, and as in the first case of the kind observed, this organ was always implicated.

The affection is frequently mistaken for a simple neuralgia of the parts, connected with some occult lesion, which the unaccustomed eye apparently detects in the normal condition of the parts.

Affections similar in some respects have been described as "neuralgia of the pharynx or larnyx." Of the latter condition, Morell McKenzie, in his classic work on "Diseases of the Throat and Nose," says: "It is apparently very rare." McKenzie had met with only nine cases, and the pain was either on the right or left side, and darted from the larynx to the ear. It was usually confined to one side; in all cases it was distinctly intermittent, and in three cases it was relieved by pressure. Eight of the cases recovered under persistent use of quinine and local sedative applications, but all were obstinate.

It attacks all persons with the same impartiality as does rheumatism, but it is more frequent in those who are subject to sudden exposure. The disease presents no anatomical characteristics, but in most cases there are circumscribed spots of slight but changeable conjestion and swelling.

In treatment, Professor INGALS had derived considerable benefit from local sedative or stimulant applications, according to the nature of the case, but had placed great reliance upon the internal remedies which are indicated in rheumatism in other parts. Of these, salicylate of soda, iodide and bromide of potassium, salol, cimicifuga, phytolacca, guiac, oil of wintergreen, and stillingia had all been tried with more or less benefit, but he had derived the best results from the extract of phytolacca, gr. III or IV, three or four times a day, with tonics and gentle laxatives, and the occasional use of iodide of potassium or the bromide of potassium, when the throat symptoms were very annoying. As local applications, he had derived most benefit from the spray of a mixture of morphia 4 grs., carbolic acid and tannic acid āā gr. xxx, glycerine and water āā oz. iv., to be applied once a day, or a weaker solution of the same applied more frequently. In some cases tincture of iodine, a 60-grain solution of nitrate of silver, or a superficial application of the galvano-cautery, has proven useful.

"The External Application of Sulphur in Sciatic Neuralgia," by J. W. Cowden, of Rock Island.

"Possibilities of Volapük, or Universal Language, in Relation to Medical Science," by David Prince, M. D., of Jacksonville.

"Digestive Ferments for Dissolving False (diphtheritic) Membrane," by Dr. A. J. Saunier.

OFFICERS FOR ENSUING YEAR.

*President*—Charles Warrington Earle, of Chicago.

*First Vice-President*—P. H. Oyler, of Mt Pulaski.

*Second Vice-President*—George L. Eyster Rock Island.

*Permanent Secretary*—D. W. Graham, of Chicago.

*Assistant Secretary*—T. M. Cullimore, of Jacksonville.

*Treasurer*—Thomas M. McIlvaine, of Peoria.

*Members of Judicial Council* (whose term of office expires in 1891)—G. Wheeler Jones, Danville; E. T. Goble, Earlville; Dr. Donaldson, Morrison; A. K. Vanhorn, Jerseyville.

STANDING COMMITTEES.

*Practice of Medicine*—F. C. Robinson, Wyanette; Elbert Wing, Chicago; J. W. Newcomer, Petersburg.

*Surgery*—L. L. McArthur, Chicago; J. S Miller, Peoria; W. W. Barritt, Onarga

*Obstetrics*—O. B. Will, Peoria; T. J. Pitner, Jacksonville; A. F. Rooney, Quincy; R. H. Engert, Chicago.

*Gynæcology*—Henry T. Byford, Chicago; C. Truesdale, Rock Island; E. P. Holderness, Chenoa.

*Drugs and Medicines*—J. L. Hallam, Centralia; W. T. Thackeray, Chicago; F. J. Parkhurst, Danvers.

*Ophthalmology and Otology*—H. M. Starkey, Chicago; J. F. Keefer, Sterling; A. E. Prince, Jacksonville.

*Necrology*—E. Ingals, Chicago; J. H. Rauch, Springfield; J. S. Whitmire, Metamora.

*Publication*—D. W. Graham, Chicago; T. M. McIlvaine, Peoria; J. W. Cullimore, Jacksonville.

*Diseases of Children*—A. T. Darrah, Bloomington; Catharine Miller, Lincoln.

SPECIAL COMMITTEES.

*Physiology*—Alfonso Wetmore, Waterloo.

*Electricity in Gynæcology*—F. H. Martin, Chicago.

*Dermatology*—R. W. Bishop, Chicago.

*Antiseptic Obstetrics*—J. B. Davison, Moline.

*Bacteriology*—Frank Billings, Frank S. Johnson, Chicago.

*Legislation for the Insane*—Clark Gapen, J. G. Kiernan, Chicago; E. P. Cook, Mendota.

*The Neuroses, Functional and Reflex*—Geo. Wheeler Jones, Danville.

*Surgery of the Joints*—A. E. Hoadley, Chicago.

*Compound Fractures*—D. A. K. Steele, Chicago.

*Uterine Hæmorrhage*—Thomas Galt, Rock Island.

*Diseases of the Alimentary Canal*—A. J. Baxter, Astoria.

*Medical and Sanitary Legislation*—B. M. Griffith, Springfield; W. A. Haskell, Alton; John L. White, Bloomington; Albert B. Strong, Chicago.

*Influence of Appreciable Meteorological and Topographical Conditions on the Prevalence of Acute Diseases*—Nathan S. Davis, John H. Hollister, James F. Todd, Chicago; Edgar P. Cook, Mendota; Geo. W. Jones, Danville.

*Biographical*—John H. Hollister, Ephraim Ingals, Chicago; Charles C. Hunt, Dixon; Francis B. Haller, Vandalia; A. M. Powell, Collinsville; Edgar P. Cook, Mendota; John S. Williams, Otterville; George W. Jones, Danville; Robert Boal, Peoria; Hugh R. Guthrie, Sparta; S. C. Plummer, Rock Island.

*Improvement of State and Local Medical Organization and on Revision of the Constitution*—Edgar P. Cook, Mendota; Thomas M. McIlvaine, Peoria; A. E. Goodwin, Rockford.

Next place of meeting, Jacksonville.

